# The Rainbow and the Umbrella of Temporomandibular Disorders—Total Antioxidant Capacity and Total Oxidant Status in Patients with Myofascial Pain with Referral

**DOI:** 10.3390/jcm14124022

**Published:** 2025-06-06

**Authors:** Joanna Kuć, Anna Zalewska, Krzysztof Dariusz Szarejko, Małgorzata Żendzian-Piotrowska, Walery Tarnawski, Sara Zięba, Mateusz Maciejczyk

**Affiliations:** 1 Department of Prosthodontics, Medical University of Bialystok, M. Skłodowskiej-Curie 24A Street, 15-276 Białystok, Poland; 2 Restorative Dentistry Department, Medical University of Bialystok, M. Skłodowskiej-Curie 24A Street, 15-276 Białystok, Poland; anna.zalewska1@umb.edu.pl (A.Z.); sara.zieba@umb.edu.pl (S.Z.); 3 Independent Laboratory of Experimental Dentistry, Medical University of Bialystok, M. Skłodowskiej-Curie 24A Street, 15-276 Białystok, Poland; 4 Private Health Care, Physical Therapy and Rehabilitation, Warszawska 79 Street, 15-201 Białystok, Poland; biuro@rehabilitacja-lecznicza.pl; 5 Department of Hygiene, Epidemiology and Ergonomics, Medical University of Bialystok, Mickiewicza 2C Street, 15-022 Białystok, Poland; malgorzata.zendzian-piotrowska@umb.edu.pl (M.Ż.-P.); mateusz.maciejczyk@umb.edu.pl (M.M.); 6 Center for Implantology and Orthodontics ‘’Royal Dental’’, Raciborska 69 Street, 44-200 Rybnik, Poland; walux@interia.pl

**Keywords:** antioxidants, myofascial pain with referral, oxidative stress, oxidative stress index, temporomandibular disorders, total antioxidant capacity, total oxidant status

## Abstract

**Background**: Temporomandibular disorders (TMDs) represent an umbrella term encompassing various musculoskeletal dysfunctions, including those affecting the masticatory muscles. This study aimed to compare the salivary levels of non-enzymatic antioxidants and redox balance between patients with temporomandibular myofascial pain with referral and matched healthy controls. **Methods**: The sample consisted of 44 individuals diagnosed with temporomandibular myofascial pain with referral and a matched control group. The procedure included a clinical examination based on the Diagnostic Criteria for Temporomandibular Disorders and saliva collection. Biochemical analysis included the assessment of reduced glutathione (GSH) levels, total antioxidant status (TAC), total oxidant status (TOS), and the oxidative stress index (OSI). **Results**: Patients with temporomandibular myofascial pain with referral exhibited higher levels of total oxidant status and glutathione. The mean value of total antioxidant capacity was lower, although the median was higher compared to the control group. No statistically significant differences were observed in the oxidative stress index between the two groups. **Conclusions**: Patients with temporomandibular myofascial pain with referral exhibit oxidative imbalance, reflected by increased salivary levels of non-enzymatic antioxidants, elevated total oxidant status, and significant differences in antioxidant capacity compared to healthy controls. Individually tailored physical activity, proper nutrition, and targeted supplementation may be necessary to maintain oxidative-antioxidant balance in this patient population.

## 1. Introduction

Temporomandibular disorders (TMDs) are an umbrella term concerning many musculoskeletal dysfunctions that involve the masticatory muscles, the temporomandibular joint, and other associated structures [1]. In the whole group of disorders, it is possible to distinguish those of muscle or joint origin. The treatment usually requires an interdisciplinary approach from various medical specializations [1]. One of the most frequent temporomandibular myogenic dysfunctions is myofascial pain with referral [2,3].

Myofascial pain with referral is characterized by the pain experienced at the site of palpation and beyond the boundary of the palpated muscles [3]. The diagnosis criterion is familiar pain induction by provocation tests, including palpation, jaw function, and jaw movement [3]. Myofascial pain reflects overlapping autonomic, motoric, and sensory symptoms that interact with both the muscles and the surrounding connective tissue, which means fascia [4]. Myofascial pain is associated with the presence of a trigger point within a taut muscle band [4]. The presence of trigger points is associated with pain, muscle spasm, sensitivity, limited range of movement, weakness, and autonomic dysfunction [5]. According to the criteria for temporomandibular disorders, myofascial pain with referral is demonstrated by simultaneous pain within masticatory muscles and referred pain, and is modified by jaw movement, function, or parafunction [2]. According to Travel and Simons, there are major and minor criteria of myofascial pain [4,6]. The major criteria include regional pain symptoms, referred pain, palpable taut muscle band, limited movement, or gentle muscle weakness. The minor criteria concern pain and local twitching response during palpation of the trigger points, and pain relief through muscle therapy [4,6]. The referred pain is modulated by central sensitization and contributes to the complexity of the disease [4].

The pathophysiology of pain is not fully understood [7]. The literature emphasizes the role of nitrosative and oxidative stress, cation signaling, and the inflammatory response [7]. Oxidative stress demonstrates excessive production of reactive oxygen and nitrogen species (ROS and RNS) that counteract the physiological role of antioxidants. The resulting oxidative imbalance is involved in the pathophysiology of many pain-related conditions [7]. The source of oxidative stress in the masticatory system is mechanical stress associated with repeated jaw movement and overuse/misuse of the muscles [8]. Excessive reactive oxygen formation at the myofascial trigger point influences pain-related TRP (transient receptor potential) channels [4]. Only TRPV1-4, TRPA1, and TRPM8 link with pain [4]. TRPV channels are sensitive to capsaicin and vanilloids [9,10]. They maintain electrolyte homeostasis and play a crucial role in various pain stimuli such as heat, pH, and pressure [9,10]. In turn, TRPA1 is prone to external factors, including severe cold, mechanical influences, noxious compounds, reactive chemicals, and endogenous cellular damage [9]. TRPM8 is involved in the sensation of cold temperatures and pain signaling [9]. ROS fluctuations in sensory neurons affect pain intensity or hyperalgesia and neuronal plasticity by modulating the excitability of respective neurons [11]. There is some evidence that oxidative stress within the trigeminal nucleus is involved in orofacial pain in rats [11]. This is gaining importance due to the fact that the trigeminal sensory nuclei complex is associated with peripheral and central mechanisms, ascending and descending pain-related pathways, and neuromodulation [12].

The original purpose of the research was to compare the salivary content of selected non-enzymatic antioxidants and oxidative-reductive balance parameters between patients with temporomandibular disorders—myofascial pain with referral and matched healthy controls. It was hypothesized that in patients with myofascial pain with referral, concentrations of non-enzymatic antioxidants and total antioxidant capacity would be lower than in the control group. In connection with this, increased total oxidant status and oxidative stress index were expected in the group with myofascial pain with referral. Bearing in mind the modern design of TMDs management, it was supposed that dietary behavior, supplementation, lifestyle factors, and physical activity may be paramount in oxidative stress modulation. It was also suggested that conscious regulation of glutathione levels based on personalized antioxidants supplementation with respect to the hormesis curve could be a novel strategy in OS management. Nevertheless, these processes may be suppressed by the antioxidant paradox.

## 2. Materials and Methods

### 2.1. Ethical Issues

The research was performed upon obtaining the consent of the Bioethics Committee of the Medical University of Bialystok, Poland (permission number: R-I-002/322/2016, date: 29 September 2016, study group sample collection; APK.002.175.2023, date: 30 March 2023, control group sample collection and biochemical determination; APK.002.248.2024, date: 18 April 2024, study group biochemical determination), in line with the tenets of the Declaration of Helsinki of the World Medical Association (WMA) and the Guidelines for Good Clinical Practice (GCP). The participation in the study was voluntary. All subjects received comprehensive information about the nature, scope, and course of clinical procedures. Each subject expressed written consent to participate in the research. At every phase of the study, the patients could decide to leave the experiment without any consequences.

### 2.2. Subjects and the Sample Size

The research was performed in the Department of Prosthodontics at the Medical University of Bialystok, Poland. The study group consisted of 44 patients (32 females, 12 males) with temporomandibular disorder—myofascial pain with referral. The subjects were diagnosed with respect to Diagnostic Criteria for Temporomandibular Disorders (DC/TMD) [13]. The average age was 23.6 ± 1.9 and ranged from 18 to 25 years. The age frame of the patients was determined by the fact that the aging process begins around the age of 30 [14], and that there exists a relationship between oxidative stress and aging [15].

Inclusion criteria:

- Diagnosis of myofascial pain with referral with respect to DC/TMD;
- Pain within the craniofacial and/or craniomandibular area (Visual analogue scale—VAS ≥ 8 points);
- Full natural dentition, class I of Angle’s Molar Classification and canine position—all considered as a criteria of normal physiological occlusion;
- No current history of orthodontic treatment or retention status above three years after completion of treatment.

Exclusion criteria:

- Injuries within the craniofacial and/or craniomandibular area;
- Any surgical treatment within the craniofacial and/or craniomandibular region;
- Any occlusal splint therapy;
- Any prosthetic treatment;
- Any physiotherapy within the craniofacial and/or craniomandibular area;
- Any diseases associated with the activity of the masticatory muscles;
- Metabolic disorders;
- Any medications, regardless of whether they have been used chronically in the past or present;
- Any individually tailored diet and taking supplements in the last six months.

The control group enrolled 44 matched healthy people (22 females, 22 males), ranging from 18 to 25 years. The average age was 22.3 ± 2.2. The subjects demonstrated a lack of pain within the craniofacial and/or craniomandibular area, including myofascial pain with referral. The subjects fulfilled all exclusion criteria.

### 2.3. Saliva Collection

The spitting method was used to collect non-stimulated saliva [16]. The material was obtained after an overnight rest, always between 8.00 and 9.00 a.m. No food or drinks were consumed by the patients. The exception was pure water drunk at least 2 h before taking saliva. All hygiene procedures in the oral cavity were also abandoned. To ensure comfortable, non-stressful conditions, the saliva was collected in the Department of Prosthodontics at the Medical University of Bialystok, Poland, in the same room where the clinical examination was conducted. Sampling was preceded by rinsing the mouth twice with distilled water at room temperature, and after at least 5 min of adaptation to environmental conditions. The patient was sitting in the dental chair. The head was slightly tilted down, with limited movement of the face and mouth. Secreted saliva was collected directly into a sterile Falcon^®^ tube (BD Biosciences, San Jose, CA, USA) and then placed in an ice bucket. The time of collection was 10 min to a maximum volume of 5 mL, with the saliva spat out in the first minute being omitted. The method was described in a previous publication [8].

A calibrated pipette with an accuracy of 100 μL was used to determine the volume of saliva, and the flow of non-stimulated saliva was obtained by dividing the saliva volume by the time of secretion. After sampling, the saliva was immediately centrifuged in constant conditions (20 min., 3000× *g*, +4 °C, MPW 35; MPW Med. Instruments, Warsaw, Poland). To protect the samples from oxidation due to their processing and storage, butylated hydroxytoluene was added to the supernatants (BHT, Sigma–Aldrich, Saint Louis, MO, USA; 10 μL 0.5 M BHT per 1 mL of saliva). For biochemical tests, saliva samples were frozen at—80 °C and then stored under these conditions until the time of analysis [8].

### 2.4. Biochemical Determination

#### 2.4.1. Non-Enzymatic Antioxidants

The concentration of reduced glutathione (GSH) was determined in duplicate samples using a colorimetric method. The measurement was based on the reduction of 5,5′-ditiobis-2 nitrobenzoic acid to 2-nitro-5-mercaptobenzoic acid under the influence of GSH contained in the assayed sample. By the wavelength of 412 nm, absorbance variations were measured, and the concentration of 2-nitro-5-mercaptobenzoic acid was calculated from a calibration curve specified for GSH solutions [17].

#### 2.4.2. Oxidation-Reduction Balance Parameters

The total oxidant status (TOS) was determined by the bichromatic method (560/800 nm) in triplicate samples. The measurement was based on the oxidation of Fe^2+^ ions to Fe^3+^ ions in the presence of oxidants in the sample. The detection of Fe^3+^ was carried out using xylenol orange. The TOS was measured from the hydrogen peroxide calibration curve and expressed as 1 μmol H_2_O_2_ Equiv, per mg protein [18].

The total antioxidant capacity (TAC) was evaluated in triplicate using a colorimetric method. The measurement is based on the neutralization of the ABTS^+^ (2,2-azino-bis-(3-ethylenbenzothiazoline-6-sulfonate cationic radical) solution surrounded by antioxidants contained in the test sample. Absorbance was measured at a wavelength of 660 nm. To determine total antioxidant capacity, the standard curve for Trolox (6-hydroxy-2,5,7,8-tetramethylchroman-2-carboxylic acid) was used [19]. Results were presented in Trolox mmol/mg of total protein. The oxidative stress index (OSI) was determined as the quotient of TOS to TAC (OSI = TOS/TAC) and was expressed in %.

### 2.5. Statistical Analysis

Statistical analysis was performed using GraphPad Prism 8 software (GraphPad Software, La Jolla, CA, USA) and G Power v.3.1.9.6 (Kiel University, Kiel, Germany). The Shapiro–Wilk test was applied to check whether the dataset is distributed along a normal distribution curve. The measures of central tendency related to the median were estimated and provided on a graph simultaneously with individual biomarker concentrations. The arithmetic mean and standard deviation were presented in the Table 1. The Mann–Whitney U test was applied to compare significant differences in the concentrations of individual biomarkers and saliva flow rate between the study group and control group, as well as in groups divided by gender. Post-hoc power analysis was conducted (*t*-test, means: Mann–Whitney test, two-tailed). Cohen’s d was used to assess the effect size. For conventional Cohen’s d, the following effect size cutoffs were accepted: 0.2 (small), 0.5 (medium), 0.8 (large). For adapted Cohen’s d, the following thresholds were recommended: 0.1 (small), 0.3 (medium), 0.7 (large) [20]. Statistical power (1-β) was calculated based on the relation of the effect size α and sample size (*n*). The sample size required to report statistically significant differences between relevant groups (at the 0.05 level) with a probability of 0.8 (80%) was specified. To evaluate interdependence between salivary biomarkers and saliva flow rate, Spearman’s correlation coefficient was used. A *p*-value of <0.05 was considered statistically significant.

## 3. Results

**Figure 1:**
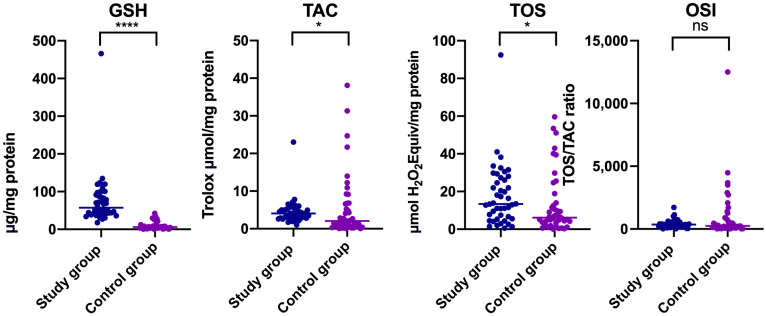
Concentrations of non-enzymatic antioxidants (GSH), total antioxidant capacity (TAC), total oxidant status (TOS), and oxidative stress index (OSI) in non-stimulated saliva in patients with temporomandibular disorder—myofascial pain with referral (*n* = 44) and control group (*n* = 44). The mean value and line at the median are given. Statistical significance: * < 0.05, **** < 0.0001, ns—nonsignificant.

The findings from this study revealed statistically significant differences in salivary concentrations of GSH, TAC, and TOS between patients who suffered from temporomandibular disorders and healthy controls (*p* < 0.05) (Table 1) (Figure 1).

**Table 1:** Concentrations of non-enzymatic antioxidants (GSH), total antioxidant capacity (TAC), total oxidant status (TOS), and oxidative stress index (OSI) in non-stimulated saliva and saliva flow rate in patients with temporomandibular disorder—myofascial pain with referral (*n* = 44) and control group (*n* = 44). The mean value, standard deviation, and median are given. With respect to post-hoc power analysis 1-β (statistical power for sample size used in present study), Cohen’s d (effect size) and *n* (sample size needed in future research for 80% test power) are presented.

Biomarkers and Saliva Flow Rate	Gender	Study Group Patients with Myofascial Pain with Referral *n* = 44			Control Group *n* = 44			Mann–Whitney *U*-Test	Power	Effect Size	Sample Size for 80% Test Power
		Mean	± SD	Median	Mean	± SD	Median	*p*	1-β	*Cohen’s* *d*	*n*
GSH	Women	71.610	31.810	69.380	11.280	12.060	6.231	<0.000 ****	1.000	2.508	9
	Men	86.240	122.000	45.550	8.966	9.169	5.974	<0.000 ****	0.654	0.893	47
	Women + Men	75.600	67.670	57.380	10.120	10.650	6.231	<0.000 ****	1.000	1.355	21
TAC	Women	4.329	1.625	4.202	6.061	10.080	1.502	0.056	0.132	0.240	594
	Men	4.857	5.805	3.022	5.020	6.699	2.457	0.403	0.051	0.026	53,221
	Women + Men	4.473	3.253	4.069	5.541	8.474	2.049	0.027 *	0.117	0.166	1190
TOS	Women	17.060	11.580	15.770	12.390	15.080	6.690	0.045 *	0.225	0.347	284
	Men	19.370	24.940	12.250	13.210	16.820	5.618	0.179	0.119	0.290	432
	Women + Men	17.690	16.030	13.350	12.800	15.790	6.130	0.015 *	0.286	0.307	351
OSI	Women	424.900	339.800	363.300	1329.000	2775.000	307.300	0.841	0.353	0.457	165
	Men	387.900	257.500	355.700	717.400	1005.000	235.700	0.763	0.220	0.449	180
	Women + Men	414.800	317.000	361.700	1023.000	2085.000	247.900	0.633	0.455	0.408	200
Saliva flow rate	Women	0.593	0.305	0.600	0.427	0.126	0.400	0.040 *	0.692	0.711	70
	Men	0.675	0.415	0.500	0.498	0.183	0.525	0.375	0.309	0.554	120
	Women + Men	0.616	0.335	0.550	0.463	0.159	0.400	0.039 *	0.752	0.583	99

Statistical significance: * < 0.05, **** < 0.0001; Cutoffs for Cohen’s d: conventional Cohen’s d: 0.2 (small), 0.5 (medium), 0.8 (large); adapted Cohen’s d for findings in temporomandibular joint research: 0.1 (small), 0.3 (medium), 0.7 (large) [20].

Higher contents of GSH and TOS were noted in patients with temporomandibular disorder—myofascial pain with referral (Median _GSH Study group_ = 57.380, Median _GSH Control group_ = 6.231; Median _TOS Study group_ = 13.350, Median _TOS Control group_ = 6.130) (Table 1). The mean value of TAC was lower in people with temporomandibular dysfunction (Mean _TAC Study group_ = 4.473, Mean _TAC Control group_ = 5.541), but median was higher compared to the control group (Median _TAC Study group_ = 4.069, Median _TAC Control group_ = 2.049) (Table 1) (Figure 1). With respect to the OSI, reported differences were not statistically significant between both groups (*p* > 0.05) (Figure 1) (Table 1).

**Figure 2:**
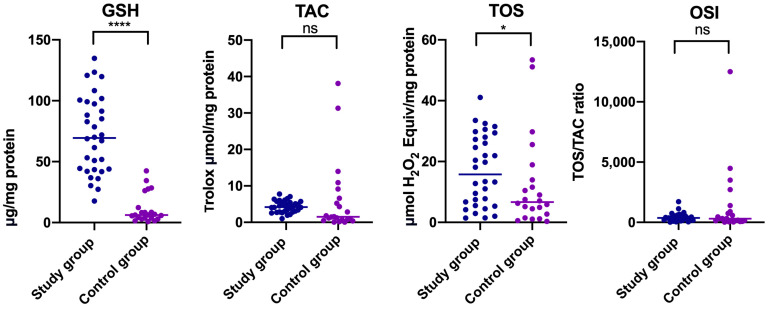
Concentrations of non-enzymatic antioxidants (GSH), total antioxidant capacity (TAC), total oxidant status (TOS), and oxidative stress index (OSI) in non-stimulated saliva in women with temporomandibular disorder—myofascial pain with referral (*n* = 32) and control group (*n* = 22). The mean value and line at the median are given. Statistical significance: * < 0.05, **** < 0.0001, ns—nonsignificant.

In relation to gender, statistically significantly higher concentrations of GSH were noted both in women and men who suffered from temporomandibular disorder compared to relevant healthy controls (Median _GSH Study group of women_ = 69.380, Median _GSH Control group of women_ = 6.231) (Table 1) (Figure 2).

**Figure 3:**
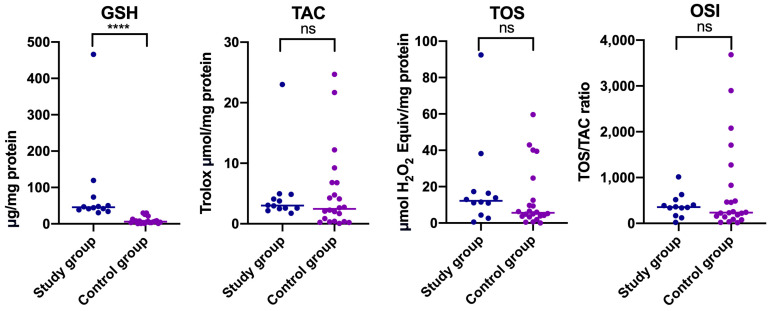
Concentrations of non-enzymatic antioxidants (GSH), total antioxidant capacity (TAC), total oxidant status (TOS), and oxidative stress index (OSI) in non-stimulated saliva in men with temporomandibular disorder—myofascial pain with referral (*n* = 12) and control group (*n* = 12). The mean value and line at the median are given. Statistical significance: **** < 0.0001, ns—nonsignificant.

A similar trend was observed in men (Median _GSH Study group of men_ = 45.550, Median _GSH Control group of men_ = 5.974) (Table 1) (Figure 3).

In the case of TOS, statistically significant differences were observed only in women (Table 1) (Figure 2 and Figure 3). Higher TOS was noted in women with myofascial pain with referral compared to relevant healthy controls (Median _TOS Study group of women_ = 15.770, Median _TOS Control group of women_ = 6.690) (Table 1) (Figure 2). No statistically significant differences in OSI and TAC levels were reported between study and control groups divided by gender (*p* > 0.05) (Table 1) (Figure 2 and Figure 3).

**Figure 4:**
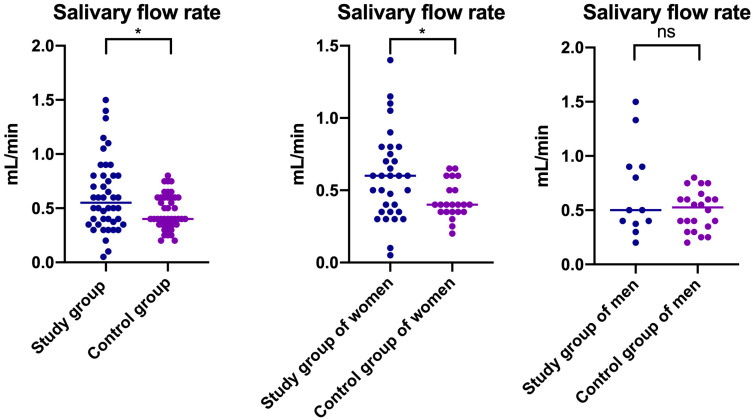
Salivary flow rate in patients with temporomandibular disorder—myofascial pain with referral (*n* = 44) and matched control group (*n* = 44) as well as in subgroup divided based on gender—group of women with myofascial pain with referral (*n* = 32), group of men with myofascial pain with referral (*n* = 12), control group of women (*n* = 22), and control group of men (*n* = 22). The mean value and line at the median are given. Statistical significance: * < 0.05, ns—nonsignificant.

Statistically significantly higher saliva flow rate was noted in the study group compared to healthy controls (Median _Saliva flow rate Study group_ = 0.550, Median _Saliva flow rate Control group_ = 0.400) (Table 1, Figure 4). A similar trend was observed in women compared to the relevant control group (Median _Saliva flow rate Study group of women_ = 0.600, Median _Saliva flow rate Control group of women_ = 0.400) (Table 1, Figure 4). No statistically significant differences in saliva flow rate were noted between the study and control groups of men (*p* > 0.05) (Table 1) (Figure 4).

**Figure 5:**
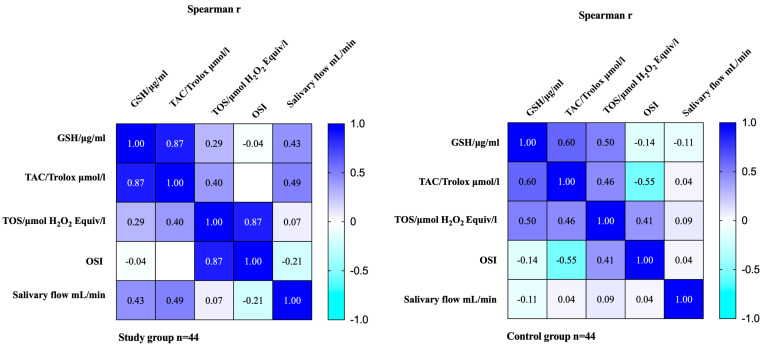
Heatmap of intercorrelations between salivary GSH, TAC, TOS, OSI, and salivary flow rate in patients with temporomandibular disorder—myofascial pain with referral (*n* = 44) and matched control group (*n* = 44). The Spearman’s correlation coefficients are given.

In patients with myofascial pain with referral, a direct proportional relationship was noted between the saliva flow rate and the salivary concentrations of GSH and TAC (r = 0.432, *p* = 0.003; r = 0.490, *p* < 0.001, respectively) (Figure 5). Statistically significant correlation was noted between TOS and TAC (r = 0.396, *p* = 0.008) (Figure 5). In the case of the control group, TOS revealed a statistically significant link with GSH, TAC, and OSI (r = 0.497, *p* = 0.001; r = 0.458, *p* = 0.002; r = 0.413, *p* = 0.005, respectively) (Figure 5).

## 4. Discussion

Oxidative stress links with many human diseases, including aging, cancer, diabetes, vascular disease, as well as dysfunction of the central and peripheral nervous system, including chronic pain [7,21,22,23]. However, there is still a literature gap regarding the role of oxidative stress in temporomandibular disorders. There are limited studies concerning the impact of oxidative stress on TMDs [24]. Current clinical and preclinical insights focus on the identification and modulation of oxidative/nitrosative stress biomarkers, which align with a patient-centered approach embracing personalized TMDs management. Temporomandibular disorders are the most common chronic orofacial pain conditions, and the ROS formation is one of the causes of TMDs [7]. Understanding the role of oxidative/nitrosative stress in TMDs pathology is of great assistance in TMDs guidance.

One of the interesting biomarkers of oxidative stress considered in this study is reduced glutathione (GSH). Glutathione is a tripeptide composed of three amino acids, such as cysteine, glutamic acid, and glycine [21,25,26]. This is the most abundant intracellular antioxidant in the body, which plays a crucial role in health and disease [25]. The concentration of GSH varies and depends on the type of tissue [26]. The average content is from 1 to 10 mM, with an increased level found in the brain from 0.5 to 3.4 μmol/g. In the central nervous system, the highest concentration of GSH occurs in glial cells of the cortex [26]. Considering different brain structures, the great absorption of GSH is found in the retina compared to the hypothalamus, spinal cord, midbrain, hippocampus, cerebellum, and cerebral cortex [26]. In the case of the retina, sun exposure leads to an excessive formation of reactive oxygen/nitrogen species, and GSH plays an important homeostatic role in these processes [27]. The brain and other tissues favor a common pathway for GSH synthesis [26]. The main functions of GSH include strengthening the immune system, prevention of cell damage caused by oxidative stress, participation in apoptosis, prostaglandin synthesis inhibition, transport facilitation of reactive metals and amino acid across membranes, metal homeostasis, DNA repair, enzyme activation, metabolism of toxins and carcinogens, myelin maturation, neuronal differentiation, and support for embryonic mammalian development [26]. Furthermore, GSH serves as a supply of extracellular cysteine and neuronal glutamate [26]. Probably, it has a binding site for glutamate receptors and may play a role as a neuromodulator/neurotransmitter [26]. GSH intensifies NMDA receptor responses and neuronal activation [26]. In vivo and in vitro decreased concentration of GSH leads to an impairment of motor neurons [26], which may potentially implicate the pathophysiology of myofascial pain.

Imbalance in GSH levels results in accelerated ageing processes and neurodegeneration [22,26]. Reduced content of glutathione is observed in autism, Alzheimer’s disease, Parkinson’s disease, schizophrenia, bipolar disorder, amyotrophic lateral sclerosis, and multiple sclerosis [22,26]. Other related pathologies concern the circulatory system, the immune system, the lungs, liver, gastrointestinal system, and metabolism [22]. Due to the fact that decreased GSH contributes to the onset and progression of neurological and neurodegenerative diseases, it may be considered as a marker for diagnostic screening for these disorders [26]. Jafri highlighted that muscle overuse seems to be the potential mechanism leading to a decrease in glutathione levels and an increase in ROS production and the appearance of myofascial trigger points [28]. This is extremely important in the context of the pathophysiology of myofascial pain and the potential tendency to clenching and grinding of the teeth. Furthermore, coexisting decreased content of GSH, exaggerated oxidative species formation, and excessive mechanical overload contribute to synovial hyperplasia within the temporomandibular joint (TMJ) [29]. TMJ inflammation also links with oxidative stress. It can be assumed that reduced GSH concentration may increase the susceptibility to TMD [29].

It should be noted that one of the causes of oxidative stress is ischemia [5]. Local ischemia can be considered as a co-player of the redox balance in patients with myofascial pain with referral. Koca et al. stressed that these processes may resemble a vicious cycle in which ischemia initiates oxidative stress, which in turn causes ischemia [5]. Local ischemia within the myofascial tissue facilitates muscle spasms and the formation of trigger points [5]. Excessive physical stress does not lead to the formation of trigger points in every individual [5]. Travel and Simons hypothesized that overloading a muscle initiates the rupture of the sarcoplasmic reticulum and increased concentration of calcium at the tissue level [5]. It promotes vasoconstriction and, consequently, ischemia [5]. In connection with this, ischemia-reperfusion phenomenon leads to local and systemic inflammatory response, leucocyte-endothelial cell adhesion, transendothelial leucocyte migration, increased platelet-leucocyte aggregation, elevated microvascular permeability, and reduced endothelium-dependent relaxation [30]. Ischemia tends to cause free radical-mediated damage [30]. The leucocytes that appeared during reperfusion promote the inflammatory cascade [30]. The role of the above-mentioned GSH in the antioxidant defense mechanism seems to be paramount [30]. These findings gain importance when considering the pathophysiology of myofascial pain with referral that overlaps stress, anxiety, depression, muscle overuse/misuse, chronic microtraumas, and vitamin and mineral deficiencies [5,31]. It should be stressed that vitamin D deficiency negatively impacts skeletal muscles through oxidative stress and mitochondrial dysfunction [32,33]. As a neuroactive steroid, vitamin D triggers neuronal excitability in the pain pathway. It means that vitamin D deficiency contributes to central neuronal hypersensitivity linked with chronic pain. Vitamin D insufficiency is considered a precipitating factor of myofascial pain syndrome [32]. In conclusion, in the case of myofascial pain, the oxidative stress seems to be both the cause and effect of the disorders.

Ege et al. revealed a lower concentration of GSH in a group with internal derangement of the temporomandibular joint (*n* = 70) compared to healthy controls (*n* = 70) [24]. Observed differences were not statistically significant (*p* = 0.069) [24]. Demir et al. reported statistically significantly lower serum levels of GSH in patients with temporomandibular joint disorders than in healthy controls [34]. Rezazadeh et al. showed statistically significant reduced salivary content of glutathione reductase in people with temporomandibular joint disorder compared to healthy controls [35]. Our study revealed the opposite results to those expected. GSH content was significantly higher in patients who suffered from myofascial pain with referral compared to the control group. This tendency was also observed in the subgroups divided based on gender (Figure 1, Figure 2 and Figure 3) (Table 1). Increased GSH may suggest an adaptive response to oxidative stress [21]. At this point, it should be mentioned that the causal link between glutathione levels and the occurrence of disease requires explanation. One of the confounding factors is the “antioxidant paradox” [21,36]. Besides antioxidant properties, GSH may trigger prooxidant hormesis activity and contribute to hormesis, affecting the body to reinforce its endogenous antioxidant defenses. In connection with this, redox balance may be the cause or consequence of the disease [21]. Hormesis represents a biological phenomenon characterized by an adaptive biphasic dose-response, which is based on stimulation that is beneficial at low doses, and inhibition or toxicity at high doses [37]. Exposure to low doses of stressors may promote or modulate physiological homeostasis, while higher doses may lead to physiological imbalance and adverse effects [37]. These dynamics are typically represented by a bell-shaped hormesis curve [38]. Hormesis may modulate biological processes at multiple levels, including the cellular, organ, individual, and population levels [37]. The most common hormetic models include dietary restriction, fasting, cold exposure, radiation, energy deficiency, chemical stress, biological factors, and nutritional hormetins [37,39,40]. All triggers can be categorized as deleterious, neutral, or beneficial stressors [41]. These contribute to the activation of hermetic mechanisms, impacting glutathione levels and mitochondrial biogenesis [41]. The glutathione system is one of the most well-established models of hormesis, initially observed in bacteria [39]. In this model, oxygen exposure and the resulting oxidative stress lead to the upregulation of nuclear factor erythroid 2-related factor 2 (Nrf2), which is associated with the synthesis of glutathione [39]. In the context of the conducted research and the observed discrepancy in GSH levels, the following questions arise: At what point on the hormesis curve are patients suffering from myofascial pain with referral located—on the ascending or descending phase? How do potential dietary hormetins, physical activity, and the biopsychosocial component of Axis II in the DC/TMD protocol influence the observed GSH levels? Do these triggers produce an additive effect, or do they interact synergistically? At which phase of the hormesis curve should antioxidant supplementation be introduced to achieve a beneficial, non-toxic effect [38]? Should patients be monitored through repeated biochemical testing in order to determine their individual position on the hormesis curve? Identifying this position may help explain the differences in glutathione levels observed between the study and control groups. Repeated biochemical assessments over time might be useful. However, an individually tailored oxidative stress profile may complicate this approach. Further research involving the implementation of controlled stressors under specific conditions is needed. Nevertheless, it should be remembered that sometimes “less is more” [39].

Achieving personalized stability in glutathione status is one of the proposed modern strategies for health promotion and disease prevention [21,39]. Minich et al. highlighted that the causal dependence between glutathione levels and the risk of illness/treatment still needs clarification [21]. This author emphasized that vitamins, minerals, and food can significantly affect circulating glutathione, which may influence clinical benefits [21]. Current strategies involve the application of food and nutrients that may elevate or maintain optimal glutathione levels [21]. Nutritional interventions focus on the role of N-acetylcysteine as a supplement for glutathione support, intake of dietary protein as the source of amino acids, omega-3 fatty acids, vitamins such as B2, B5, B12, C, E, alpha-lipoic acid, selenium, curcumin, and green tea [21,27]. Special role may be attributed to the plant foods that contain glutathione, e.g., asparagus, avocado, cucumber, brassica vegetables, green beans, spinach, and papaya [21,27]. The art of eating is gaining importance. Plant-based food and practical strategies for “eating the rainbow” seem to be paramount [42]. The rainbow strategy is distinguished by nutrition criteria: red foods and inflammation (I), orange foods and reproductive health (II), yellow foods and digestion (III), green foods and cardiovascular health (IV), blue-purple foods and cognition (V) [42]. Bearing in mind the pathophysiology of temporomandibular myofascial pain described in previous [8,31,43] and current publications, all these groups of plant-based foods should be incorporated in an individually tailored novel approach in TMD management. Nevertheless, it should be mentioned that the rainbow strategy may be associated with certain limitations. Immediate painful response associated with compression of the periodontal ligament and delayed painful response determined by hyperalgesia—both linked with orthodontic force application [44], and analogously with teeth “overclenching”—may limit the rainbow strategy implementation, especially in the case of eating hard, raw plant-based food. The help of dieticians may be essential in developing a nutritional strategy. Summarizing, dietary behavior focused on antioxidant-rich foods may reduce the risk of noncommunicable disease by elevating total antioxidant capacity (TAC). Studies on subjects suffering from oxidative stress conditions revealed that 70% of interventions associated with plant foods and supplements resulted in elevated plasma/serum TAC levels [45]. Lifestyle factors affecting the salivary TAC levels include exercise, smoking habits, alcohol dependence, as well as occupational exposure to toxicants [45]. Oral behavior also seems to be important. Bearing in mind the fact that in a healthy person the contact of the teeth should be about 17.5 min per day [46], and that probably in people with temporomandibular disorders this time is prolonged [47], it can be assumed that the TAC level will simultaneously decrease as a response to repetitive mandibular movements associated with clenching or grinding of the teeth. This is a kind of analogy to the excessive overload of the muscles mentioned above. More research is needed. Special attention in TAC modulation may be attributed to cellphones associated with exposure to radio frequency radiation and watching TV [45]. It was found that emotional and psychological factors may influence salivary TAC levels. In connection with this, watching a cheerful, comical video for 30 min contributes to an increase in salivary TAC levels [45].

Our study revealed that mean TAC levels in people with temporomandibular myofascial pain with referral were lower than in matched healthy controls, but the median value was conversely higher (Table 1) (Figure 1). The lower average TAC in the study group was caused by the presence of outliers in the control group (Figure 1). With respect to gender, declared differences were not statistically significant (Table 1) (Figure 2 and Figure 3). Statistically significant increased TOS was observed in people who suffered from temporomandibular dysfunction compared to the control group (Table 1) (Figure 1). A similar tendency was observed in a subgroup of women (Table 1) (Figure 2). Elevated TOS was reported in women with myofascial pain than in relevant healthy controls. No statistically significant differences were stated with respect to OSI between the study group and control group, and in subgroups divided based on gender, respectively. Statistically significant correlation was noted between TOS and TAC in patients with myofascial pain with referral (Figure 5). In the case of the control group, TOS correlated with GSH, TAC, and OSI (Figure 5).

Lawaf et al. reported no statistically significant differences in salivary TAC between people who suffered from TMDs with and without pain, and healthy controls [48]. In turn, plasma TAC was significantly lower in both TMD patients compared to the control group [48]. Ozcan-Kucuk et al. revealed that TAC levels in patients with sleep bruxism were lower than those of matched healthy controls. The TOS and OSI were higher in people with sleep bruxism than in the control group [49]. Madariaga et al. reported statistically significantly lower content of TOS in people with myogenous temporomandibular disorder than in healthy subjects [50]. TAC levels were higher in the TMD group compared to the control group. There were no statistically significant differences in TAC and TOS levels between a group of myalgia and a group of myofascial pain [50]. Ege et al. reported statistically significantly lower TAC and higher TOS in people with internal derangement compared to matched controls [24]. The latest research highlighted interdependence between sleep bruxism intensity and low TAC, high AOPP (advanced oxidative protein products), and TBARS (thiobarbituric acid-reacting substances) serum levels [51].

Koca et al. revealed a statistically significant inversely proportional relationship between the visual analogue scale concerning VAS and TAC in people with general myofascial pain in the body [5]. Simultaneously, a direct proportional link was reported between VAS and TOC as well as the OSI [5]. This interdependence between TAC, TOS, OSI, and pain scores may be integrated to specify the severity of the dysfunction, including antioxidant system depletion, as well as to monitor antioxidant treatment [5]. It should be noted that in our study, one of the many inclusion criteria was craniofacial/craniomandibular pain equal to and above 8 points of the VAS. Other studies revealed an inversely proportional relationship between TAC and TOS in patients with general myofascial pain dysfunction and direct proportional dependence between TAC and pressure pain thresholds (PPT) [52]. This study also revealed the opposite results to those expected with respect to VAS. There was no correlation between VAS scores and TAC, so pain evaluation with respect to PPT seemed to provide more sensitive data [52].

Our study revealed a direct proportional relationship between the saliva flow rate and the salivary concentrations of GSH and TAC in people with myofascial pain with referral. These connections were not reported in the case of the control group (Figure 4). Salivary flow rates change depending on the circadian cycle, individual hydration, food stimulation, and oral hygiene [53]. It should be noted that a higher saliva flow rate was observed in the study group as well as in women with myofascial pain with referral compared to the healthy controls (Figure 5).

Similarly to the abovementioned GSH, TAC levels may be modulated by nutritional and supplement interventions. Caffeinated or alcoholic antioxidant beverages elevate salivary TAC by 50% and urinary TAC by 40% in the intervention [45]. Vitamin C and vitamin E may also influence the salivary and urinary TAC [45]. Red wine extract contributes to an increase in salivary TAC within 30 min [45]. A significant role may be attributed to blackberry juice, spinach, strawberries, and fruit-based drinks [45]. In turn, physical stress reduces salivary TAC [45].

Buczko et al. revealed that orthodontic treatment may modify the oxidant-antioxidant balance in the saliva of clinically healthy subjects [54]. This author reported that 2 years after orthodontic intervention, the total antioxidant status was lower as compared to the values noted before and one week after the treatment [54]. OSI and TOS were the highest after one week of the treatment. Similar values of OSI were reported before and after 2 years of interventions. With respect to TOS, the lowest values were observed after 2 years of treatment [54]. According to this, it would be extremely interesting to assess the impact of occlusal splints on the oxidative stress biomarkers profile in patients with TMDs. Vrbanovic et al. stated that in women, occlusal splint contributes to elevating the capacity to remove free radicals. This author emphasized that data concerning TAC levels in TMDs are inconsistent. Many studies reported lower TAC levels in patients with TMDs compared to the control group. Vrbanovic et al. suggest that TAC levels may be related to the chronicity of the disorder [55]. It means that chronic pain lasting 6 months or more provides an opportunity to adapt to potentially high oxidant levels, resulting in higher TAC levels. It reflects a compensatory reaction to a distorted oxidative balance [55].

Oxidative stress and associated changes in signaling pathways have multiple pathophysiological consequences at different stages of life [56]. To mitigate the negative effects and toxicity of oxidative stress on health, systematic physical activity should be implemented to reduce the noxious effects of free radicals. Exercises contribute to ROS formation and affect the level of muscle contraction. Modest ROS accumulation increases muscle strength [56]. However, during intense exercises, its excessive production leads to acute muscle fatigue [56]. Bearing in mind the pathophysiology of myofascial pain with referral, a necessity to provide proper hygiene of the circulatory system with respect to the discharge muscle metabolites as well as due to oxidative-antioxidant balance, individually tailored physical activity and modern eating behavior should be implemented as a novel strategy in TMDs management.

Bearing in mind the literature gap, our study contributes to the expansion of knowledge concerning oxidative stress biomarkers in the TMD umbrella evolvement. The main advantage of this research is a strictly defined protocol that remains in line with Diagnostic Criteria for Temporomandibular Disorders (DC/TMD) [57]. Once again, we highlighted the role of nutrition and supplementation as a key to understanding the oxidative-antioxidative balance disorders, the pathophysiology and etiology of TMDs, including myofascial pain with referral. We stressed the importance of individually tailored eating behavior in TMD patients exposed to oxidative stress. Based on the literature, we pointed out the risk of lifestyle and diet-related diseases associated with oxidative stress, which may also affect our study group. The main limitation was the inequality of the study group in terms of gender, with the predominance of the number of women, with an associated imbalance in the number of men. Therefore, it is advised to be cautious with the results of this study. More research is needed. The next restriction was limiting the interpretation of the results due to the lack of consideration of the exact nutritional profile of the patients and people from the control group. Future research should take this into account.

## 5. Conclusions

1. Patients with temporomandibular myofascial pain with referral exhibit oxidative imbalance, as evidenced by increased salivary concentrations of the non-enzymatic antioxidant glutathione, elevated total oxidative status, and statistically significant differences in total antioxidant capacity compared to the control group.
2. Despite the observed alterations in individual markers of oxidative stress (TAC, TOS), no statistically significant differences were found in the oxidative stress index between patients with temporomandibular myofascial pain with referral and the control group.
3. Patients with temporomandibular disorders—specifically myofascial pain with referral may benefit from individually tailored physical activity, proper nutrition and eating behaviors, as well as targeted supplementation, in order to maintain oxidative-antioxidant balance. The “rainbow strategy” could play a key role in this approach.

## Data Availability

The article contains complete data used to support the findings of this study.

## Abbreviations

The following abbreviations are used in this manuscript:

TMDs	Temporomandibular disorders
ROS	Reactive oxygen species
RNS	Reactive nitrogen species
DC/TMD	Diagnostic Criteria for Temporomandibular Disorders
VAS	Visual analogue scale
GSH	Reduced glutathione
TOS	The total oxidant status
TAC	The total antioxidant capacity
OSI	The oxidative stress index

## References

[1] Brighenti N., Battaglino A., Sinatti P., Abuín-Porras V., Sánchez Romero E.A., Pedersini P., Villafañe J.H. (2023). Effects of an interdisciplinary approach in the management of temporomandibular disorders: A scoping review. Int. J. Environ. Res. Public Health.

[2] Schiffman E., Ohrbach R., Truelove E., Look J., Anderson G., Goulet J.-P., List T., Svensson P., Gonzalez Y., Lobbezoo F. (2014). Diagnostic criteria for temporomandibular disorders (DC/TMD) for clinical and research applications: Recommendations of the International RDC/TMD Consortium Network and Orofacial Pain Special Interest Group. J. Oral Facial Pain Headache.

[3] Kapos F.P., Exposto F.G., Oyarzo J.F., Durham J. (2020). Temporomandibular disorders: A review of current concepts in aetiology, diagnosis and management. Oral Surg..

[4] Widyadharma I.P.E. (2021). The role of oxidative stress, inflammation and glial cell in pathophysiology of myofascial pain. Adv. Psychiatry Neurol./Postępy Psychiatr. Neurologii.

[5] Koca İ., Tutoglu A., Boyacı A., Pehlivan Y., Yıldız H., Turkbeyler I., Sarıcicek E., Taysi S., Onat A.M. (2014). An evaluation of oxidative stress and antioxidant capacity in patients with myofascial pain syndrome. Mod. Rheumatol..

[6] Duarte F.C., West D.W., Linde L.D., Hassan S., Kumbhare D.A. (2021). Re-examining myofascial pain syndrome: Toward biomarker development and mechanism-based diagnostic criteria. Curr. Rheumatol. Rep..

[7] Gianò M., Franco C., Castrezzati S., Rezzani R. (2023). Involvement of Oxidative Stress and Nutrition in the Anatomy of Orofacial Pain. Int. J. Mol. Sci..

[8] Kuć J., Szarejko K.D., Maciejczyk M., Dymicka-Piekarska V., Żendzian-Piotrowska M., Zalewska A. (2025). Oxidative imbalance as a co-player in jaw functional limitations and biopsychosocial profile in patients with temporomandibular disorder—Myofascial pain with referral. Front. Neurol..

[9] Zhang M., Ma Y., Ye X., Zhang N., Pan L., Wang B. (2023). TRP (transient receptor potential) ion channel family: Structures, biological functions and therapeutic interventions for diseases. Signal Transduct. Target. Ther..

[10] Seebohm G., Schreiber J.A. (2021). Beyond hot and spicy: TRPV channels and their pharmacological modulation. Cell Physiol. Biochem..

[11] Khan S., Tao F. (2025). Mechanisms for Orofacial Pain: Roles of Immunomodulation, Metabolic Reprogramming, Oxidative Stress and Epigenetic Regulation. Biomedicines.

[12] Iwata K., Takeda M., Oh S.B., Shinoda M., Farah C., Balasubramaniam R., McCullough M. (2017). Neurophysiology of orofacial pain. Contemporary Oral Medicine.

[13] Ohrbach R., Gonzalez Y., List T., Michelotti A., Schiffman E. (2014). Diagnostic Criteria for Temporomandibular Disorders (DC/TMD) Clinical Examination Protocol. www.rdc-tmdinternational.org.

[14] Chalise H.N. (2019). Aging: Basic concept. Am. J. Biomed. Sci. Res..

[15] Ebert T., Tran N., Schurgers L., Stenvinkel P., Shiels P.G. (2022). Ageing–oxidative stress, PTMs and disease. Mol. Asp. Med..

[16] Navazesh M. (1993). Methods for collecting saliva. Ann. N. Y. Acad. Sci..

[17] Moron M.S., Depierre J.W., Mannervik B. (1979). Levels of glutathione, glutathione reductase and glutathione S-transferase activities in rat lung and liver. Biochim. Biophys. Acta (BBA)-Gen. Subj..

[18] Erel O. (2005). A new automated colorimetric method for measuring total oxidant status. Clin. Biochem..

[19] Erel O. (2004). A novel automated direct measurement method for total antioxidant capacity using a new generation, more stable ABTS radical cation. Clin. Biochem..

[20] Zieliński G., Gawda P. (2025). Defining effect size standards in temporomandibular joint and masticatory muscle research. Med. Sci. Monit. Int. Med. J. Exp. Clin. Res..

[21] Minich D.M., Brown B.I. (2019). A review of dietary (phyto) nutrients for glutathione support. Nutrients.

[22] Vázquez-Meza H., Vilchis-Landeros M.M., Vázquez-Carrada M., Uribe-Ramírez D., Matuz-Mares D. (2023). Cellular compartmentalization, glutathione transport and its relevance in some pathologies. Antioxidants.

[23] Pizzorno J. (2014). Glutathione!. Integr. Med. Clin. J..

[24] Ege B., Kucuk A.O., Koparal M., Koyuncu I., Gonel A. (2021). Evaluation of serum prolidase activity and oxidative stress in patients with temporomandibular joint internal derangement. CRANIO^®^.

[25] Sekhar R.V. (2021). GlyNAC Supplementation improves glutathione deficiency, oxidative stress, mitochondrial dysfunction, inflammation, aging hallmarks, metabolic defects, muscle strength, cognitive decline, and body composition: Implications for healthy aging. J. Nutr..

[26] Iskusnykh I.Y., Zakharova A.A., Pathak D. (2022). Glutathione in brain disorders and aging. Molecules.

[27] Giustarini D., Milzani A., Dalle-Donne I., Rossi R. (2023). How to increase cellular glutathione. Antioxidants.

[28] Jafri M.S. (2014). Mechanisms of myofascial pain. Int. Sch. Res. Not..

[29] Melis M., Di Giosia M. (2016). The role of genetic factors in the etiology of temporomandibular disorders: A review. Cranio^®^.

[30] Jefferies H., Coster J., Khalil A., Bot J., McCauley R.D., Hall J.C. (2003). Glutathione. ANZ J. Surg..

[31] Kuć J., Szarejko K.D., Gołębiewska M. (2020). Evaluation of soft tissue mobilization in patients with temporomandibular disorder-myofascial pain with referral. Int. J. Environ. Res. Public Health.

[32] Channarong P., Phongamwong C. (2023). Prevalence and risk factors of vitamin D deficiency among patients with chronic myofascial pain syndrome: A cross-sectional study. BMC Nutr..

[33] Piriyaprasath K., Kakihara Y., Hasegawa M., Iwamoto Y., Hasegawa Y., Fujii N., Yamamura K., Okamoto K. (2024). Nutritional Strategies for Chronic Craniofacial Pain and Temporomandibular Disorders: Current Clinical and Preclinical Insights. Nutrients.

[34] Demir C.Y., Kocak O.F., Bozan N., Ersoz M.E., Demir H. (2018). Is there a role for oxidative stress in temporomandibular joint disorders?. J. Oral Maxillofac. Surg..

[35] Rezazadeh F., Fassihi N., Mahdavi D., Tabesh A., Khorami E.T. (2022). Evaluation Salivary Level of Glutathion Reductase, Catalase and Free Thiol in Patients with Temporomandibular Joint Disorder. https://assets-eu.researchsquare.com/files/rs-1552798/v1/43c734cf-ec3f-4428-b3ca-04857e254618.pdf?c=1674996276I.

[36] Rahal A., Kumar A., Singh V., Yadav B., Tiwari R., Chakraborty S., Dhama K. (2014). Oxidative stress, prooxidants, and antioxidants: The interplay. BioMed Res. Int..

[37] Wan Y., Liu J., Mai Y., Hong Y., Jia Z., Tian G., Liu Y., Liang H., Liu J. (2024). Current advances and future trends of hormesis in disease. npj Aging.

[38] Radak Z., Ishihara K., Tekus E., Varga C., Posa A., Balogh L., Boldogh I., Koltai E. (2017). Exercise, oxidants, and antioxidants change the shape of the bell-shaped hormesis curve. Redox Biol..

[39] Schirrmacher V. (2021). Less can be more: The hormesis theory of stress adaptation in the global biosphere and its implications. Biomedicines.

[40] Rattan S., Watson R.R., Preedy V.R. (2013). Nutritional hormetins and ageing. Bioactive Food as Dietary Interventions for the Aging Population.

[41] Lee D.-H., Jacobs D.R. (2015). Hormesis and public health: Can glutathione depletion and mitochondrial dysfunction due to very low-dose chronic exposure to persistent organic pollutants be mitigated?. J. Epidemiol. Community Health.

[42] Minich D.M. (2019). A review of the science of colorful, plant-based food and practical strategies for “Eating the Rainbow”. J. Nutr. Metab..

[43] Szarejko K.D., Gołębiewska M., Lukomska-Szymanska M., Kuć J. (2023). Stress Experience, Depression and Neck Disability in Patients with Temporomandibular Disorder—Myofascial Pain with Referral. J. Clin. Med..

[44] Negruțiu B.-M., Vaida L.L., Judea-Pusta C., Romanec C., Moca A.E., Costea C.P., Staniș C.-E., Rus M. (2024). Orthodontic pain and dietary impact considering age groups: A comparative study. J. Clin. Med..

[45] Peluso I., Raguzzini A. (2016). Salivary and urinary total antioxidant capacity as biomarkers of oxidative stress in humans. Pathol. Res. Int..

[46] Kumar L. (2014). Biomechanics and clinical implications of complete edentulous state. J. Clin. Gerontol. Geriatr..

[47] Antonelli J., Hottel T.L., Siegel S.C., Brandt R., Silva G. (2013). The occlusal guard: A simplified technique for fabrication and equilibration. Gen. Dent..

[48] Lawaf S., Azizi A., Tabarestani T. (2015). Comparison of serum and salivary antioxidants in patients with temporomandibular joint disorders and healthy subjects. J. Dent..

[49] Ozcan-Kucuk A., Ege B., Koparal M., Gonel A., Koyuncu I. (2021). Evaluation of the oxidative stress level and serum prolidase activity in patients with sleep bruxism. Comb. Chem. High Throughput Screen..

[50] Madariaga V.I., Jasim H., Ghafouri B., Ernberg M. (2021). Myogenous temporomandibular disorders and salivary markers of oxidative stress—A cross-sectional study. J. Oral Rehabil..

[51] Fulek M., Frosztega W., Wieckiewicz M., Szymanska-Chabowska A., Gac P., Poreba R., Mazur G., Sciskalska M., Kepinska M., Martuszewski A. (2025). The link between sleep bruxism and oxidative stress based on a polysomnographic study. Sci. Rep..

[52] Etoz O.A., Ataoglu H., Erel O., Celik H., Herken E.N., Bayazit Y.A. (2009). Association of serum total antioxidant capacity and total oxidant status with pain perception in patients with myofacial pain dysfunction. Int. J. Neurosci..

[53] Kamodyová N., Tóthová L.u., Celec P. (2013). Salivary markers of oxidative stress and antioxidant status: Influence of external factors. Dis. Markers.

[54] Buczko P., Knaś M., Grycz M., Szarmach I., Zalewska A. (2017). Orthodontic treatment modifies the oxidant–antioxidant balance in saliva of clinically healthy subjects. Adv. Med. Sci..

[55] Vrbanović E., Lapić I., Rogić D., Alajbeg I. (2019). Changes in salivary oxidative status, salivary cortisol, and clinical symptoms in female patients with temporomandibular disorders during occlusal splint therapy: A 3-month follow up. BMC Oral Health.

[56] Simioni C., Zauli G., Martelli A.M., Vitale M., Sacchetti G., Gonelli A., Neri L.M. (2018). Oxidative stress: Role of physical exercise and antioxidant nutraceuticals in adulthood and aging. Oncotarget.

[57] Osiewicz M., Ciapała B., Bolt K., Kołodziej P., Więckiewicz M., Ohrbach R. (2024). Diagnostic criteria for temporomandibular disorders (DC/TMD): Polish assessment instruments. Dent. Med. Probl..

